# Facilitating time series classification by linear law-based feature space transformation

**DOI:** 10.1038/s41598-022-22829-2

**Published:** 2022-10-27

**Authors:** Marcell T. Kurbucz, Péter Pósfay, Antal Jakovác

**Affiliations:** 1 Department of Computational Sciences, Wigner Research Centre for Physics, 29-33 Konkoly-Thege Miklós Street, Budapest, 1121 Hungary; 2 Institute of Data Analytics and Information Systems, Corvinus University of Budapest, 8 Fővám Square, Budapest, 1121 Hungary

**Keywords:** Computational science, Statistics

## Abstract

The aim of this paper is to perform uni- and multivariate time series classification tasks with linear law-based feature space transformation (LLT). First, LLT is used to separate the training and test sets of instances. Then, it identifies the governing patterns (laws) of each input sequence in the training set by applying time-delay embedding and spectral decomposition. Finally, it uses the laws of the training set to transform the feature space of the test set. These calculation steps have a low computational cost and the potential to form a learning algorithm. For the empirical study of LLT, a widely used human activity recognition database called AReM is employed. Based on the results, LLT vastly increases the accuracy of traditional classifiers, outperforming state-of-the-art methods after the proposed feature space transformation is applied. The fastest error-free classification on the test set is achieved by combining LLT and the k-nearest neighbor (KNN) algorithm while performing fivefold cross-validation.

## Introduction

In machine learning and data mining, time series classification (TSC) is one of the most challenging tasks^1,2^; it aims to assign all unlabeled time series to one of the predefined categories. TSC is widely studied across various domains, including activity recognition^3–6^, biology^7–10^, and finance^11–14^, but its growing popularity is primarily due to the rapidly increasing amount of temporal data collected by widespread sensors^15^. Accordingly, with the proliferation of the Internet of Things and big data technologies, TSC has become increasingly crucial. A TSC task can be defined as a uni-^16,17^ or multivariate^18,19^ problem, depending on whether one or more values are observed at a particular point in time. In recent decades, a number of methods have been proposed to address both problems and can be divided into feature-based and distance-based approaches (see, e.g.,^20^). The main difference between the two is that the former approach contains a feature extraction phase before the classification stage, while the latter performs classification based on a suitable (dis)similarity measure of the series. Hereinafter, we focus only on the methods and algorithms of the feature-based approach that are used during the feature extraction and classification phases.

Some methods for feature extraction include basic statistical methods^21–23^ and spectral-based methods, such as discrete Fourier transform (DFT)^24^ or discrete wavelet transform (DWT)^25,26^ methods, in which features of the frequency domain are considered. Others are based on singular value decomposition (SVD)^27^, where eigenvalue analysis is applied to the dimensional reduction of the feature set. Furthermore, there are some model-based methods that are mainly used to capture information about the dynamic behavior of the investigated series. Within this group, the different versions of the autoregressive (AR) integrated moving average (ARIMA) model^28^ are widely applied (see, e.g.,^5,29–31^). In most of these works, the coefficients of a fitted AR model are applied as features^30,31^, or they are used to build a more complex generative model^29^.

After the time series data are transformed into feature vectors, they are classified by conventional classifiers such as logistic regression (LR)^32,33^, decision tree (DT)^34^, random forest (RF)^35^, k-nearest neighbor (KNN)^36^, support vector machine (SVM)^37,38^, relevance vector machine (RVM)^39^, and Gaussian process (GP)^40^ classifiers. Alternatively, deep neural networks (DNNs) can be applied to automatically compile the feature space before classification^41–43^.

The aim of this paper is to find a solution for uni- and multivariate TSC tasks with a linear law-based feature space transformation (LLT) approach that can be applied between the feature extraction and classification phases. LLT is based on an idea borrowed from physics, the so-called existence of conserved quantities. In general, these are quantities that remain constant for the whole system if some basic conditions are fulfilled. The conservation of energy and momentum are perhaps the most known examples, but in theory infinitely many conserved quantities can be constructed for a given system, although not all of them represent new information. LLT searches for conserved quantities in time series data and characterizes them with vectors (hereinafter referred to as laws).

To facilitate TSC tasks, LLT, which is a data-driven approach, identifies a set of laws related to each class and then applies these sets to transform the time series data of a new instance. Laws related to each class compete to reveal the class of the new time series; however, only the laws corresponding to the correct class can transform it close to the null vector. In essence, this transformation generates a new feature space utilizing the linear laws belonging to classes. The resulting features are expected to be more selective for the given TSC task. Similarly to principal component analysis (PCA)^44,45^, LLT is based on spectral decomposition. However, in contrast to PCA, LLT focuses on the most invariant property of the time series data instead of the most variable property.

In practice, first, LLT separates the training and test sets of the instances. Then, it identifies the governing patterns (laws) of each input sequence in the training set by applying time-delay embedding and spectral decomposition. Finally, it uses the laws of the training set to transform the feature space of the test set. These calculation steps have a low computational cost and the potential to form a learning algorithm.

Generally, raw time series contain various types of distortion, which makes their comparison and classification difficult^46^. The common types of distortion are noise, offset translation, amplitude scaling, longitudinal scaling, linear drift, and discontinuities^47^. In addition to its low computational cost, the main advantage of LLT is that it is robust to noise and amplitude scaling by definition. Discontinuities, provided they rarely occur, do not alter the resulting laws significantly. Offset transformation and linear drift can also be easily adapted, and the laws are very generalizable, i.e., if the training set contains instances with specific offset or linear drift values, the laws will be able to treat any others. Furthermore, it becomes more and more resistant to the other distortions mentioned above as the number (not the rate) of (atypical) instances increases in the training set. The reason is that the transformation is applied by the most suitable law per class. Thus, if the training set contains instances with a similar set of distortions as the new instance, the transformation is applied based on their laws. For the same reason, the generated feature set, and thus any learning algorithm based on it, is largely immune to catastrophic forgetting^48–50^ when the training set is extended.

For the empirical study of LLT, a widely used human activity recognition database called AReM is applied. This database contains temporal data from a wireless sensor network worn by users who were asked to perform seven different activities. The related TSC task is the identification of these activities based on the noisy sensor data generated by the users. Based on the results, the accuracy of traditional classifiers are greatly increased using LLT, outperforming state-of-the-art methods after the proposed feature space transformation is applied. Due to its robustness against a significant amount of noise (which is a characteristic of the HAR databases^51^), fast and error-free classification is achieved based on the combination of the KNN algorithm. (The working paper of this study is available at SSRN^52^).

The rest of this paper is organized as follows. In the Methods section, the general TSC problem as well as the LLT algorithm are introduced. In the Experimental setup section, the AReM database and the applied classification algorithms are presented. In the “Results and discussion” section, the results of the classification obtained with and without LLT are compared and discussed. Finally, in the Conclusions and future work section, conclusions are provided, and future research directions are discussed.

## Methods

### Time series classification problem

The TSC problem discussed in this paper is based on the following data structure. We consider input data as ${\varvec{X}} = \{ {\varvec{X}}_t \;\vert \; t\in \{1,2,\dots ,k\}\}$ sets (time series), where *t* denotes the observation times. The internal structure of these input data is ${\varvec{X}}_t = \{ {\varvec{x}}_t^{i,j} \;\vert \; i\in \{1,2,\dots ,n\},~j\in \{1,2,\dots ,m\}\}$, where *i* identifies the instances and *j* indexes the different input series belonging to a given instance. The output is a vector ${\varvec{y}} \in \{1,2,\dots ,c\}$ identifying the classes (*c*) of instances (${\varvec{y}} = \{ y^{i}\in {\mathbb {R}}\;\vert \; i\in \{1,2,\dots ,n\}\}$). Our goal is to predict the ${\varvec{y}}$ values (classes) from the ${\varvec{X}}$ input data.

For the sake of transparency, the above-mentioned data structure is illustrated in Table 1.

**Table 1:** Internal data structure of the TSC problem.

***i***	Input 1	Input 2	$\cdots$	Input m	Output
1	${\varvec{x}}^{1,1}_{t}$	${\varvec{x}}^{1,2}_{t}$	$\cdots$	${\varvec{x}}^{1,m}_{t}$	$y^1$
2	${\varvec{x}}^{2,1}_{t}$	${\varvec{x}}^{2,2}_{t}$	$\cdots$	${\varvec{x}}^{2,m}_{t}$	$y^2$
$\vdots$	$\vdots$	$\vdots$	$\vdots$	$\vdots$	$\vdots$
*n*	${\varvec{x}}^{n,1}_{t}$	${\varvec{x}}^{n,2}_{t}$	$\cdots$	${\varvec{x}}^{n,m}_{t}$	$y^n$

### Linear laws of time series

Before we describe how to transform the original feature space to facilitate the TSC task, the concept of linear laws^53^ is introduced. Here, we only summarize the most important results needed to understand the logic behind LLT. The detailed derivations and proofs can be found in^54^. First, let us consider a generic time series ${\varvec{z}}_t$, where $t\in \{1,2,...,k \}$ denotes the time. The $l{\text {th}}$ order ($l \in {\mathbb {Z}}^+$ and $l<k$) time-delay embedding^55^ of the given series is obtained as follows:

1 $$\begin{aligned} {\varvec{A}}=\left( \begin{matrix}{\varvec{z}}_{1}&{}{\varvec{z}}_{2}&{}\cdots &{}{\varvec{z}}_{l}\\ \ {\varvec{z}}_{2}&{}\ddots &{}\ddots &{}\vdots \\ \vdots &{}\ddots &{}\ddots &{}\vdots \\ {\varvec{z}}_{k-l}&{}\cdots &{}\cdots &{}{\varvec{z}}_{k}\\ \end{matrix}\right) . \end{aligned}$$

A linear function that maps the rows of the matrix ${\varvec{A}}$ to zero is called a (linear) law. An exact linear law can be represented by a vector ${\varvec{v}}$ that fulfills the following condition:

2 $$\begin{aligned} \Vert {\varvec{A}} {\varvec{v}} \Vert = 0. \end{aligned}$$

It can be seen that using normal quadratic norm finding ${\varvec{v}}$ is equivalent to finding the eigenvector corresponding to the zero eigenvalue of the symmetric matrix ${\varvec{S}}$, which is defined as^54^:

3 $$\begin{aligned} {\varvec{S}}={\varvec{A}}^\intercal {\varvec{A}}. \end{aligned}$$

This method can be thought of as complementary to PCA, where we are looking for the largest eigenvalues (representing the largest change in the data). Here, we search for the smallest (preferably zero) eigenvalue, which represents the direction in which the data change the least. In practice, the linear law, ${\varvec{v}}$, maps the learning set close to zero:

4 $$\begin{aligned} {\varvec{S}} {\varvec{v}} \approx {\mathbf {0}}, \end{aligned}$$

where ${\mathbf {0}}$ is a null column vector with *l* elements, ${\varvec{v}}$ is a column vector with *l* elements and ${\varvec{v}} \ne {\mathbf {0}}$. To find the ${\varvec{v}}$ coefficients of Eq. (4), first, we perform eigendecomposition on the ${\varvec{S}}$ matrix. Then, we select the eigenvector that is related to the smallest eigenvalue. Finally, we apply this eigenvector as ${\varvec{v}}$ coefficients, and hereinafter, we refer to it as the linear law of ${\varvec{z}}_t$.

### Linear law-based feature space transformation

To facilitate the classification task presented in the time series classification problem section, we transform the original feature space using linear laws.

First, instances (*i*) are divided into training ($tr\in \{1,2,\dots ,\tau \}$) and test ($te\in \{\tau +1,\tau +2,\dots ,n \}$) sets in such a way that the classes of instances are balanced in the two sets. For the sake of transparency, we assume that the original arrangement of the instances in the dataset (see Table 1) satisfies this condition for the *tr* and *te* sets. Then, we calculate the linear law (see ${\varvec{v}}$ in Eq. 4) of all input series in the training set (${\varvec{x}}^{1,1}_t,{\varvec{x}}^{2,1}_t,\dots ,{\varvec{x}}^{\tau ,m}_t$), which results in $\tau \times m$ laws (eigenvectors) in total. To do this, we create an ${\varvec{A}}^{tr,j}$ matrix for all instances in the training set (*tr*) and all input series (*j*). We calculate a separate ${\varvec{S}}^{tr,j}$ matrix for each component of this index pair (see Eqs. 1–3). Then, we use the eigenvectors related to their smallest eigenvalues as laws (see Eq. 4). As a result of these steps, the dataset presented in Table 2 is obtained.

**Table 2:** Linear laws of the training set.

Source	${\varvec{x}}^{1,1}_t$	${\varvec{x}}^{2,1}_t$	$\cdots$	${\varvec{x}}^{\tau ,1}_t$	${\varvec{x}}^{1,2}_t$	$\cdots$	${\varvec{x}}^{\tau ,m}_t$
Laws	${\varvec{v}}^{1,1}$	${\varvec{v}}^{2,1}$	$\cdots$	${\varvec{v}}^{\tau ,1}$	${\varvec{v}}^{1,2}$	$\cdots$	${\varvec{v}}^{\tau ,m}$

We remark that the linear laws (eigenvectors) contained by Table 2 are column vectors with *l* elements. For the sake of transparency, we introduce ${\varvec{V}}^j$ matrices with $l \times \tau$ dimensions that group linear laws based on the related input series (${\varvec{V}}^j = [{\varvec{v}}^{1,j},{\varvec{v}}^{2,j},\dots ,{\varvec{v}}^{\tau ,j}]$). Although we have not indexed which input series belongs to which class, because the instances belonging to each class are balanced in the *tr* and *te* sets, they are also balanced in the ${\varvec{V}}^j$ matrices. Consequently, the laws contained in each ${\varvec{V}}^j$ matrix are derived from instances that cover all classes together (${\varvec{V}}^j = \{{\varvec{V}}^j_{1},{\varvec{V}}^j_{2},\dots ,{\varvec{V}}^j_{c}\}$, where ${\varvec{V}}^j_{c}$ denotes the laws of the training set related to the input series *j* and the class *c*).

As a next step, we calculate ${\varvec{S}}^{te,j}$ matrices (see Eq. 3) from the input series of the test instances in the test set. For one instance, *m* matrices are calculated (one for each input series, see Table 3).

**Table 3:** ${\varvec{S}}$ matrices of the test set.

Source	${\varvec{x}}^{\tau +1,1}_t$	${\varvec{x}}^{\tau +2,1}_t$	$\cdots$	${\varvec{x}}^{n,1}_t$	${\varvec{x}}^{\tau +1,2}_t$	$\cdots$	${\varvec{x}}^{n,m}_t$
Matrices	${\varvec{S}}^{\tau +1,1}$	${\varvec{S}}^{\tau +2,1}$	$\cdots$	${\varvec{S}}^{n,1}$	${\varvec{S}}^{\tau +1,2}$	$\cdots$	${\varvec{S}}^{n,m}$

We left-multiply the ${\varvec{V}}^j$ matrices obtained from the training set by the ${\varvec{S}}^{te,j}$ matrices of the test set related to the same input variable (${\varvec{S}}^{\tau +1,1}{\varvec{V}}^1,{\varvec{S}}^{\tau +1,2}{\varvec{V}}^2,\dots ,{\varvec{S}}^{n,m}{\varvec{V}}^m$). Each of these products results in a matrix that inherits the dimensions of the ${\varvec{V}}^j$ matrices ($l \times \tau$). In this step, the laws contained in the ${\varvec{V}}^j$ matrices provide an estimate of whether the ${\varvec{S}}^{te,j}$ matrices of the test set are related to the same class as them. More specifically, only those columns of the ${\varvec{S}}^{te,j}{\varvec{V}}^j$ matrices are close to the null vector, and they have a relatively small variance, where the classes of the corresponding training and testing data match.

Then, we reduce the dimension of the resulting matrices by using an *f* function. This function selects the column vectors with the smallest variance from the ${\varvec{S}}^{te,j}{\varvec{V}}^j$ matrices for each class as follows:

5 $$\begin{aligned} \begin{gathered} {\varvec{S}}^{te,j}{\varvec{V}}^j=\{{\varvec{S}}^{te,j}{\varvec{V}}^j_{1},{\varvec{S}}^{te,j}{\varvec{V}}^j_{2},\dots ,{\varvec{S}}^{te,j}{\varvec{V}}^j_{c}\}, \\ f({\varvec{S}}^{te,j}{\varvec{V}}^j)=\{{\varvec{o}}^{te,j}_{1},{\varvec{o}}^{te,j}_{2},\dots ,{\varvec{o}}^{te,j}_{c}\}, \end{gathered} \end{aligned}$$

For example, the series ${\varvec{o}}^{te,j}_{c}$ is a column vector of the ${\varvec{S}}^{te,j}{\varvec{V}}^j_{c}$ matrix that has the minimum variance. Thus, after this step, the transformed feature space of the test set has $((n-\tau ) l) \times (m c)$ dimensions without the output variable (see Table 4).

**Table 4:** Feature space of the test set generated based on the linear laws of the training set.

*te*	Input 1	Input 2	$\cdots$	Input m	Output
$\tau +1$	$f({\varvec{S}}^{\tau +1,1}{\varvec{V}}^1)$	$f({\varvec{S}}^{\tau +1,2}{\varvec{V}}^2)$	$\cdots$	$f({\varvec{S}}^{\tau +1,m}{\varvec{V}}^m)$	$y^{\tau +1}$
$\tau +2$	$f({\varvec{S}}^{\tau +2,1}{\varvec{V}}^1)$	$f({\varvec{S}}^{\tau +2,2}{\varvec{V}}^2)$	$\cdots$	$f({\varvec{S}}^{\tau +2,m}{\varvec{V}}^m)$	$y^{\tau +2}$
$\vdots$	$\vdots$	$\vdots$	$\cdots$	$\vdots$	$\vdots$
*n*	$f({\varvec{S}}^{n,1}{\varvec{V}}^1)$	$f({\varvec{S}}^{n,2}{\varvec{V}}^2)$	$\cdots$	$f({\varvec{S}}^{n,m}{\varvec{V}}^m)$	$y^{n}$

Finally, to facilitate cross-validation and performance measurements, we calculate the arithmetic mean and variance of each transformed time series (see the $g_1$ and $g_2$ functions in Eq. 6). Both cases result in $m \times c$ scalars for each instance of the test set (one scalar for each input series of each class). This final transformation reduces the dimension of the $f({\varvec{S}}^{te,j}{\varvec{V}}^j)$ matrices (see Eq. 5) as follows:

6 $$\begin{aligned} \begin{gathered} g_1(f({\varvec{S}}^{te,j}{\varvec{V}}^j))=\{\text {Mean}({\varvec{o}}^{te,j}_{1}),\text {Mean}({\varvec{o}}^{te,j}_{2}),\dots ,\text {Mean}({\varvec{o}}^{te,j}_{c})\}, \\ g_2(f({\varvec{S}}^{te,j}{\varvec{V}}^j))=\{\text {Var}({\varvec{o}}^{te,j}_{1}),\text {Var}({\varvec{o}}^{te,j}_{2}),\dots ,\text {Var}({\varvec{o}}^{te,j}_{c})\}. \end{gathered} \end{aligned}$$

These means and variances are used as features to predict the class of instances in the test set (*te*). That is, the classes are predicted based on $\{\text {Mean}({\varvec{o}}^{te,1}_{1}),\text {Mean}({\varvec{o}}^{te,1}_{2}),\dots ,\text {Mean}({\varvec{o}}^{te,1}_{c}),\text {Mean}({\varvec{o}}^{te,2}_{1}),\dots ,\text {Mean}({\varvec{o}}^{te,m}_{c})\}$ in the first case and $\{\text {Var}({\varvec{o}}^{te,1}_{1}),\text {Var}({\varvec{o}}^{te,1}_{2}),\dots ,\text {Var}({\varvec{o}}^{te,1}_{c}),\text {Var}({\varvec{o}}^{te,2}_{1}),\dots ,\text {Var}({\varvec{o}}^{te,m}_{c})\}$ in the second.

In summary, we applied the linear laws of the training set to transform the feature space of test instances to facilitate their classification. If the instances of classes show similarity, the transformed test instances belonging to the same class will have values close to zero in the same places since the laws related to the same class will transform them close to null vectors (see Eq. 4).

## Experimental setup

This section describes the employed database and presents the classifier algorithms applied to the original and transformed feature spaces.

### Human activity recognition dataset

In this paper, the database called the Activity Recognition system based on Multisensor data fusion (AReM)^56^ is employed (freely available at: https://archive.ics.uci.edu/ml/datasets/Activity+Recognition+system+based+on+Multisensor+data+fusion+%28AReM%29, retrieved: 20 September 2022).

This database was compiled by using three wireless beacons that implement the IEEE 802.15.4 standard. These beacons were attached to a user’s chest and both ankles, and the user performed seven different activities, including cycling, lying down, sitting, standing, and walking, as well as two types of bending (${\varvec{y}}\in \{1,2,...,7 \}$). The signal strength of the beacons, which was measured in a unit called the received signal strength (RSS), decreased in proportion to the distance between the beacons and the applied wireless scanners.

The RSS values of these beacons were sampled at a frequency of 20 Hz. Every 50 ms, the scanner recorded an RSS value from each of the three sensors. Finally, the mean and variance of the RSS values accumulated every 250 ms were computed for each beacon, resulting in 6 features (time series; $m=6$). Every user performed a specific activity for 2 min, and 480 consecutive values were generated for each of these series ($k=480$). With the exception of the two different types of bending, 15 such datasets were recorded from each activity performed by different users. From the type 1 and type 2 bending activities, 7 and 6 datasets were collected, respectively ($n=88$).

The multivariate TSC task associated with the AReM database is to predict the type of activities based on the 6 features generated by the three wireless beacons. Before attempting to solve this task, we randomly divide the 88 instances ($5\times 15 + 7 + 6$ datasets) into training (*tr*) and test (*te*) sets. After this step, approximately $53.4\%$ of the instances belonging to each activity are included in the test set, while all other instances are part of the training set. Within each category, the test set contained the following data (see the AReM^56^ database):

- Bending 1: 2, 3, 4, 6.
- Bending 2: 2, 3, 5.
- Cycling: 1, 2, 5, 7, 9, 10, 14, 15.
- Lying: 1, 2, 6, 9, 11, 12, 14, 15.
- Sitting: 4, 7, 8, 9, 10, 11, 12, 15.
- Standing: 2, 3, 6, 7, 8, 12, 13, 14.
- Walking: 1, 3, 4, 7, 8, 9, 11, 13.

### Applied classifiers

After transforming the feature space of the test data by using the linear laws of the training set (see the linear law-based feature space transformation and human activity recognition dataset sections), we compare the accuracy and calculation time of four different classifiers on both the original and the transformed feature spaces. The applied algorithms are the ensemble, KNN, DT, and SVM algorithms, which are used with fivefold cross-validation and 30-step Bayesian hyperparameter optimization. The classification task was performed in the Classification Learner App of MATLAB (details of the applied methods and the classification process can be found at https://www.mathworks.com/help/stats/classificationlearner-app.html, retrieved: 20 September 2022).

## Results and discussion

From the original six features contained in the AReM database, first, we generated a new feature space with 42 features by using the LLT algorithm (6 features per class). To facilitate cross-validation and performance measurements, we calculated the mean and variance of each transformed time series (see Eq. 6). The means and variances in the transformed case were calculated from the 30 long time series. We then solved the classification problem of mean values and variances based on both the original and transformed feature spaces by using four different classifiers (see the “Applied classifiers” section). The results of the calculations are shown in Table 5 (for the sake of comparison, in the nontransformed case, we performed calculations based on the mean and variance of each input series of the original feature space).

**Table 5:** Classification results .

(a) Based on mean values					(b) Based on variances				
	Applied feature space					Applied feature space			
	Original		Transformed by LLT			Original		Transformed by LLT	
	Accuracy (%)	Time (s)	Accuracy (%)	Time (s)		Accuracy (%)	Time (s)	Accuracy (%)	Time (s)
Ensemble	74.5	150.7	100.0	135.3	Ensemble	68.1	172.2	91.5	213.4
KNN	85.1	54.4	100.0	42.5	KNN	80.9	63.8	100.0	59.7
DT	74.5	28.8	87.2	39.0	DT	57.4	43.6	85.1	44.6
SVM	83.0	121.2	89.4	151.2	SVM	80.9	149.9	91.5	195.2

Remarks: fivefold cross-validation and 30-step Bayesian hyperparameter optimization were applied. The total number of observations ($n-\tau$) was 47 in all four cases. In the LLT case, the calculation was based on the absolute value of the means—see subfigure (a). Confusion matrices and the details of hyperparameter optimization can be found in the Supplementary information. The calculations were performed using a computer with an Intel Core i7-6700K CPU processor running at 4.00 GHz with 16 GB RAM.

Table 5 shows that the accuracy of the original feature space was approximately $78.1\%$ on average ($79.3\%$ for the mean and $76.8\%$ for the variance), while after the transformation, we obtained approximately $93.1\%$ accurate classification ($93.1\%$ and $92.0\%$, respectively). This increase in performance was associated with only a relatively small increase in training time ($9.8\%$ on average). Furthermore, the fastest (42.5 s and 59.7 s, respectively) error-free classifications were achieved by combining the LLT and KNN algorithms, which outperformed the state-of-the-art methods. Furthermore, with this algorithm, we obtained a shorter calculation time in the case of the transformed feature space than in the original case. Moreover, the hyperparameters of this algorithm conspicuously converged very rapidly to their optimum (see the Supplementary information), which may further decrease the required optimization time.

In comparison with the recently published results related to the AReM database, Vydia and Sasikumar (2022)^57^ achieved a maximum classification accuracy of $99.63\%$ by using the DWT along with the entropy features from empirical mode decomposition (EMD) and four different classifiers. While their method is superior to several state-of-the-art machine learning techniques^57^, it is slightly less accurate than the combination of the LLT algorithm with the KNN or ensemble classifiers.

An additional advantage of the LLT algorithm is that it has a low computational cost—the transformation of the original feature space took only approximately 1.8 sec (the calculations were performed using a computer with an Intel Core i7-6700K CPU processor running at 4.00 GHz with 16 GB RAM). Additionally, LLT has the potential to form a learning algorithm by continuously improving the set of laws applied during the transformation.

## Conclusions and future work

In this paper, the LLT algorithm, which aims to facilitate uni- and multivariate TSC tasks, was introduced. LLT has a low computational cost and the potential to form a learning algorithm. For its empirical study, we applied a widely used multisensor-based human activity recognition dataset called AReM. Based on the results, LLT vastly increased the accuracy of traditional classifiers, which outperformed state-of-the-art methods after the proposed feature space transformation. The fastest error-free classification on the test set was achieved by the combination of the LLT and KNN algorithms while performing 5-fold cross-validation and 30-step Bayesian hyperparameter optimization. In this case, the hyperparameters conspicuously converged very rapidly to their optimum, which may further decrease the required optimization time.

Our future research will focus on the application of the proposed feature space transformation for portfolio selection and hearth disease classification based on ECG signals. Additionally, we will develop R and Python packages to facilitate the use of LLT. Further studies could also focus on how LLT can be applied as a part of a learning algorithm in which the set of laws used for the feature space transformation is continuously improved. Finally, it may be worthwhile to examine how LLT can be integrated into the framework of neural networks.

## Supplementary Information

Supplementary Information.

## Data Availability

The Activity Recognition system based on Multisensor data fusion (AReM)^56^ is freely available at: https://archive.ics.uci.edu/ml/datasets/Activity+Recognition+system+based+on+Multisensor+data+fusion+%28AReM%29, retrieved: 20 September 2022.
