# Supplementary material for: Facilitating time series classification by linear law-based feature space transformation
Source: Sci Rep. 2022 Oct 27;12:18026. doi: 10.1038/s41598-022-22829-2 (PMC9613694; doi:10.1038/s41598-022-22829-2)
Supplement: Supplementary file 1 — Supplementary Information. [file 41598_2022_22829_MOESM1_ESM.pdf]

Supplementary Information for Facilitating time series classification by linear law-based feature space transformation

Marcell T. Kurbucz[a,b,*], Péter Pósfay[a], Antal Jakovác[a]

[a] *Department of Computational Sciences, Wigner Research Centre for Physics, 29-33 Konkoly-Thege Miklós Street, H-1121 Budapest, Hungary*
[b] *Institute of Data Analytics and Information Systems, Corvinus University of Budapest, 8 Fővám Square, H-1093, Hungary*

Abstract

The aim of this paper is to perform uni- and multivariate time series classification tasks with linear law-based feature space transformation (LLT). First, LLT is used to separate the training and test sets of instances. Then, it identifies the governing patterns (laws) of each input sequence in the training set by applying time-delay embedding and spectral decomposition. Finally, it uses the laws of the training set to transform the feature space of the test set. These calculation steps have a low computational cost and the potential to form a learning algorithm. For the empirical study of LLT, a widely used human activity recognition database called AReM is employed. Based on the results, LLT vastly increases the accuracy of traditional classifiers, outperforming state-of-the-art methods after the proposed feature space transformation is applied. The fastest error-free classification on the test set is achieved by combining LLT and the k-nearest neighbor (KNN) algorithm while performing 5-fold cross-validation.

Keywords: Time series classification, Linear law, Feature space transformation, Feature engineering, Human activity recognition, Artificial intelligence

---

Contents

Optimization results 2
Confusion matrices 10

*Corresponding author: Tel.: +36 1 392 2222;
Email addresses: kurbucz.marcell@wigner.hu (Marcell T. Kurbucz), posfay.peter@wigner.hu (Péter Pósfay), jakovac.antal@wigner.hu (Antal Jakovác)

Figure S1: Optimization results

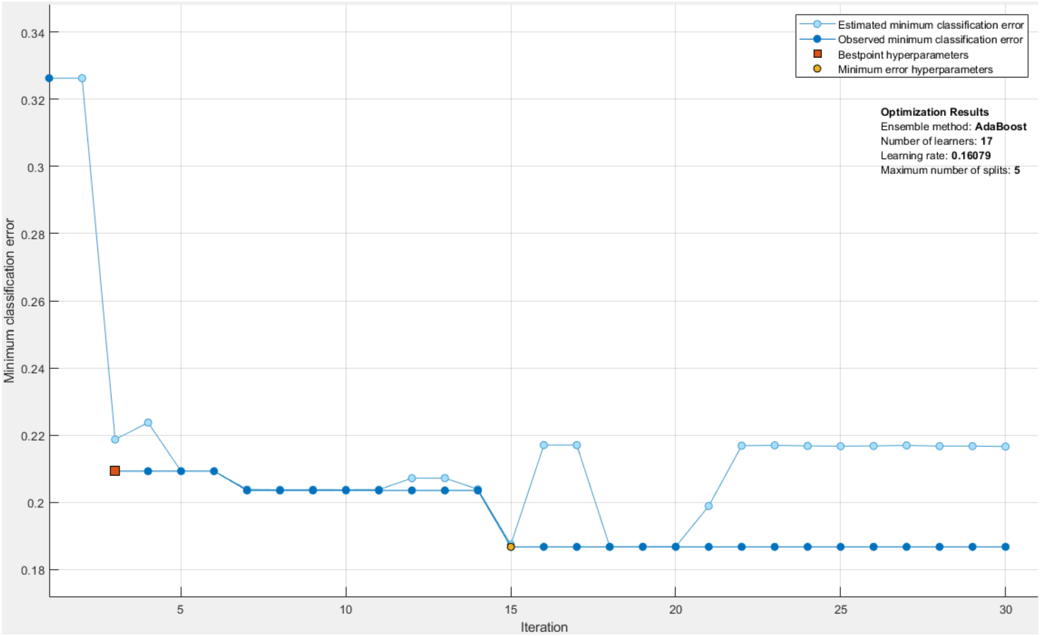

(a) Ensemble, mean, original fs.

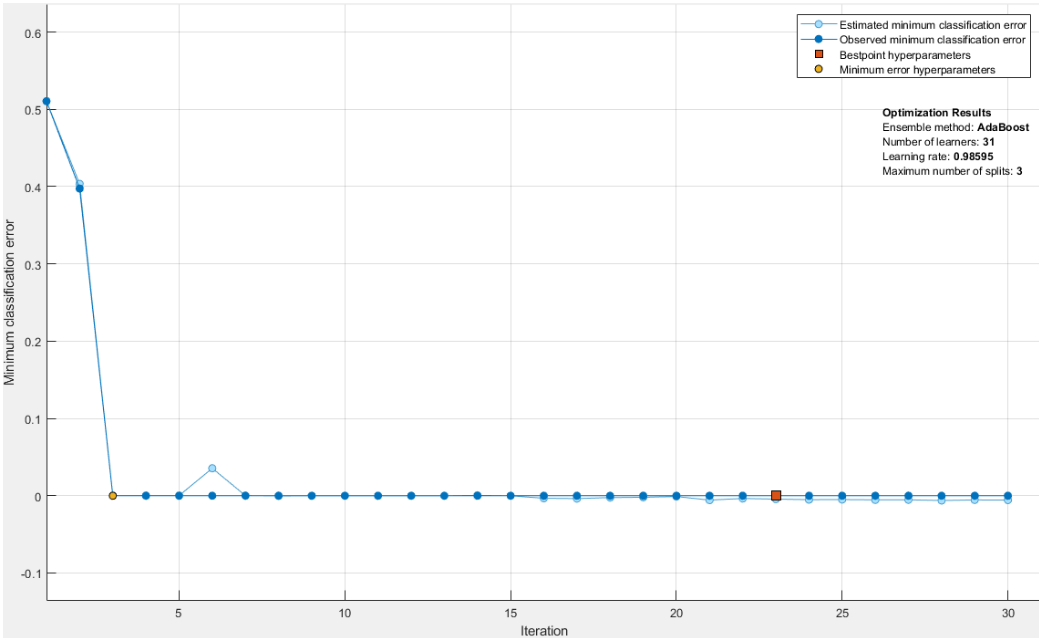

(b) Ensemble, mean, LLT-based fs.

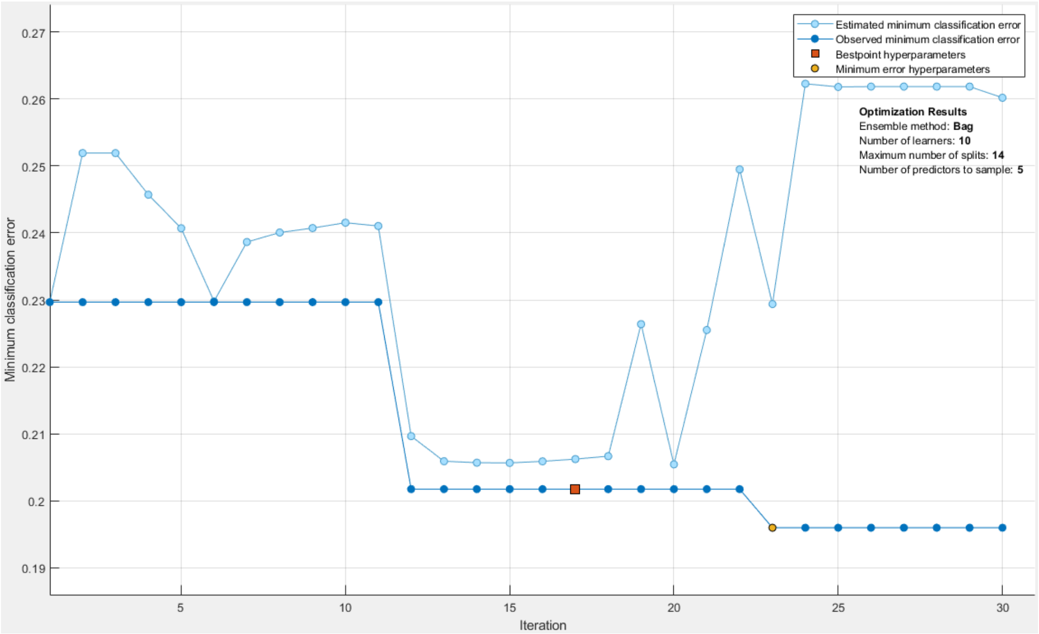

(c) Ensemble, variance, original fs.

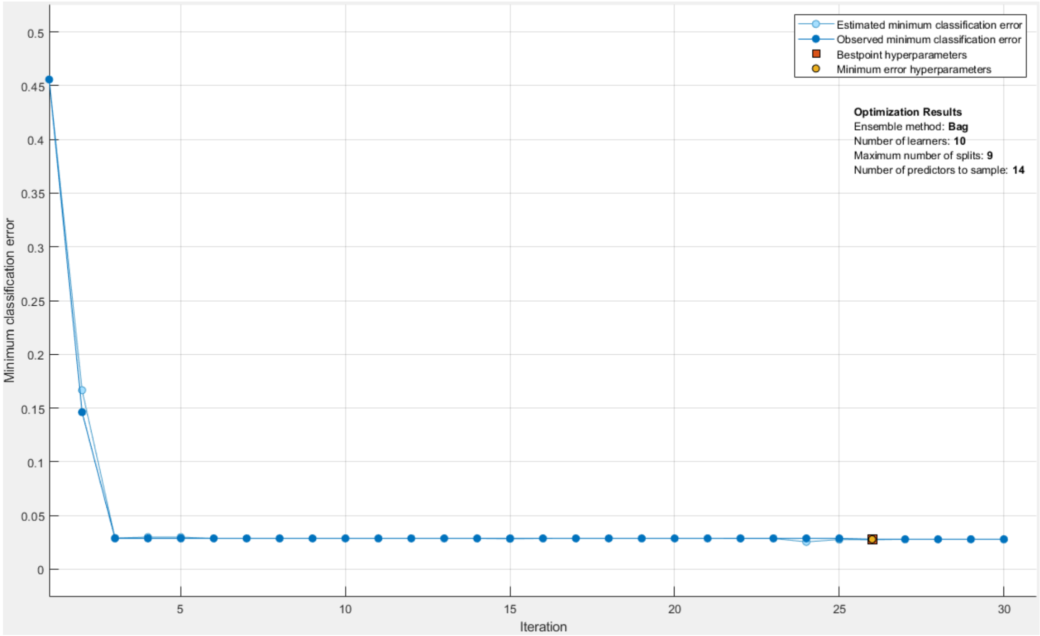

(d) Ensemble, variance, LLT-based fs.

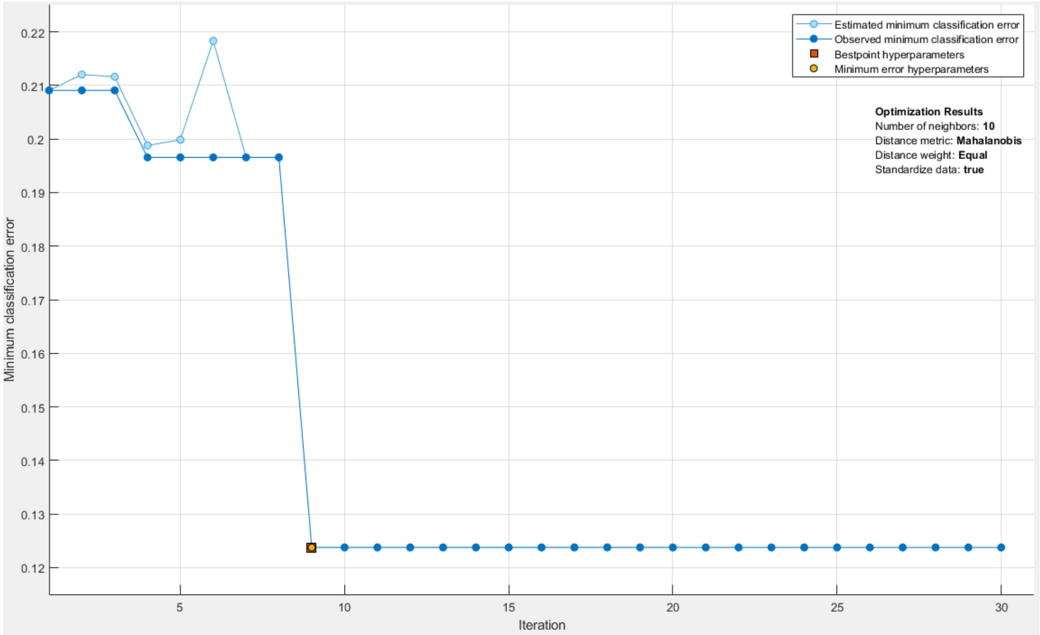

(e) KNN, mean, original fs.

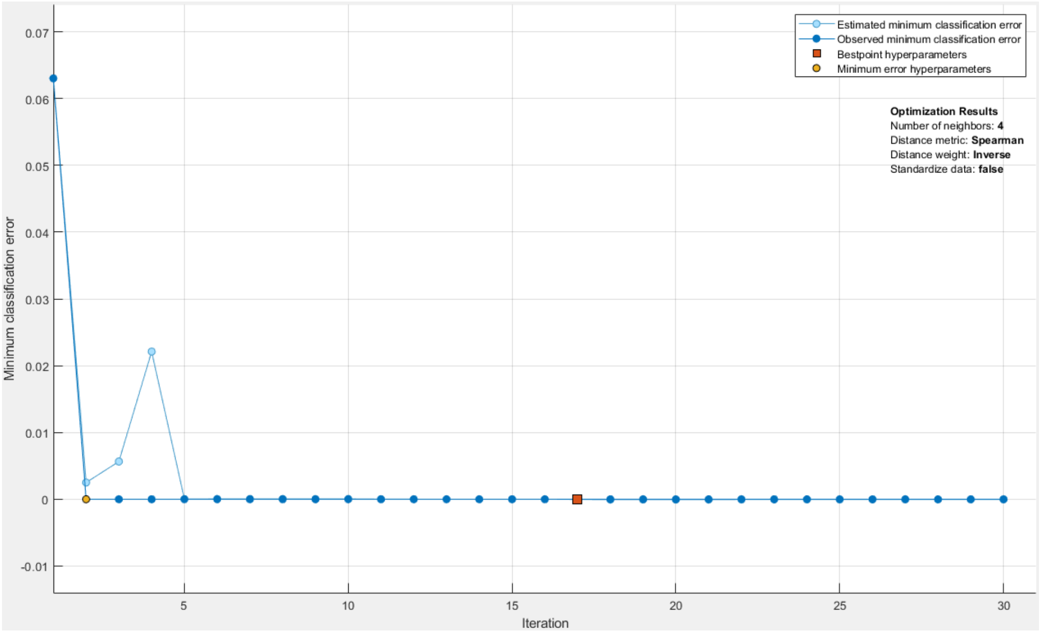

(f) KNN, mean, LLT-based fs.

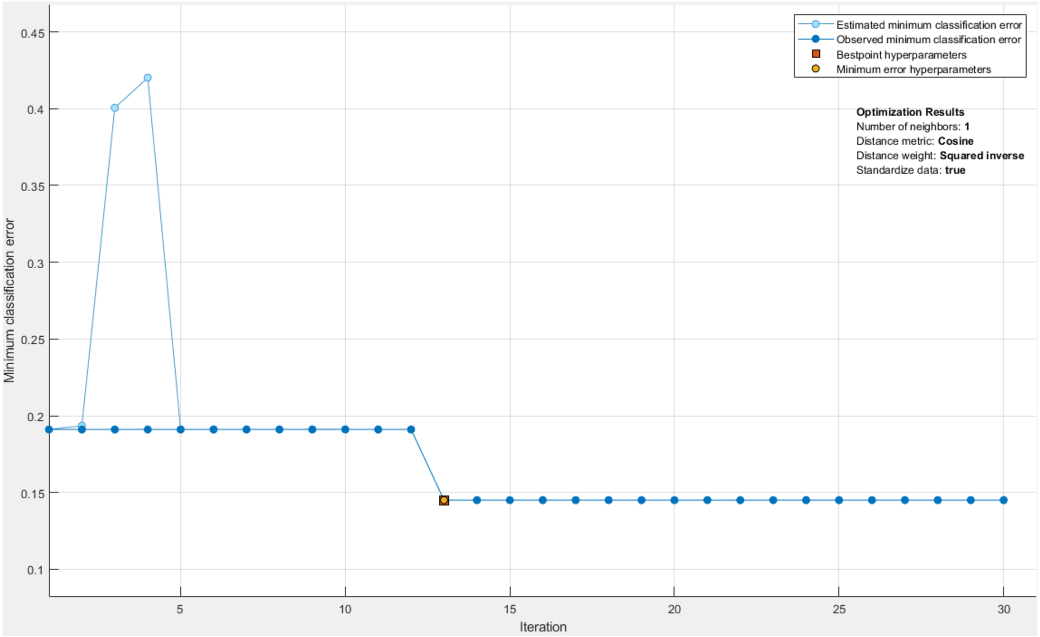

(g) KNN, variance, original fs.

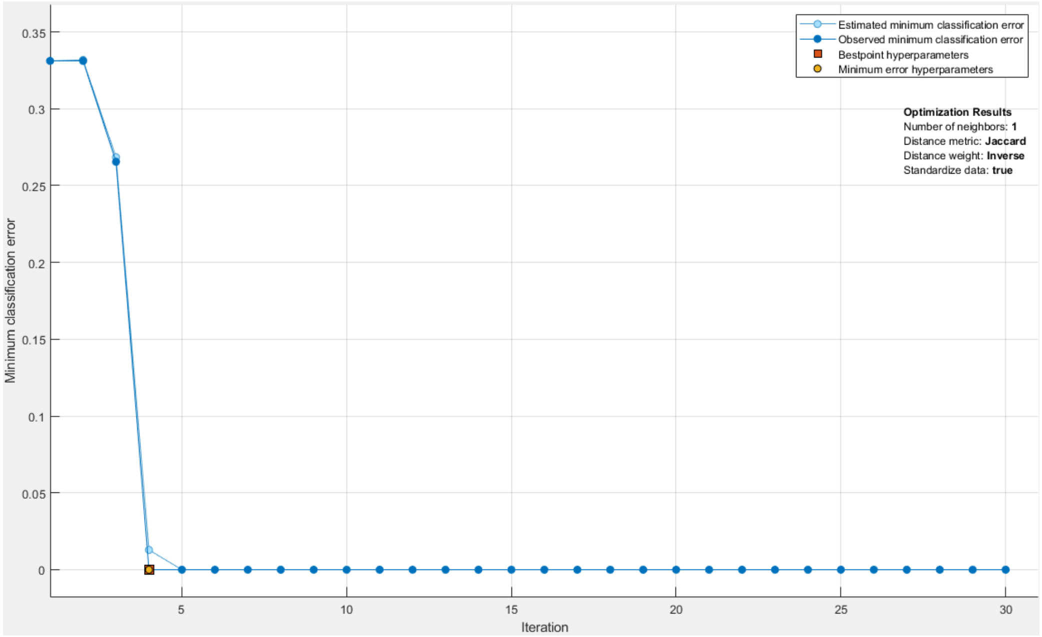

(h) KNN, variance, LLT-based fs.

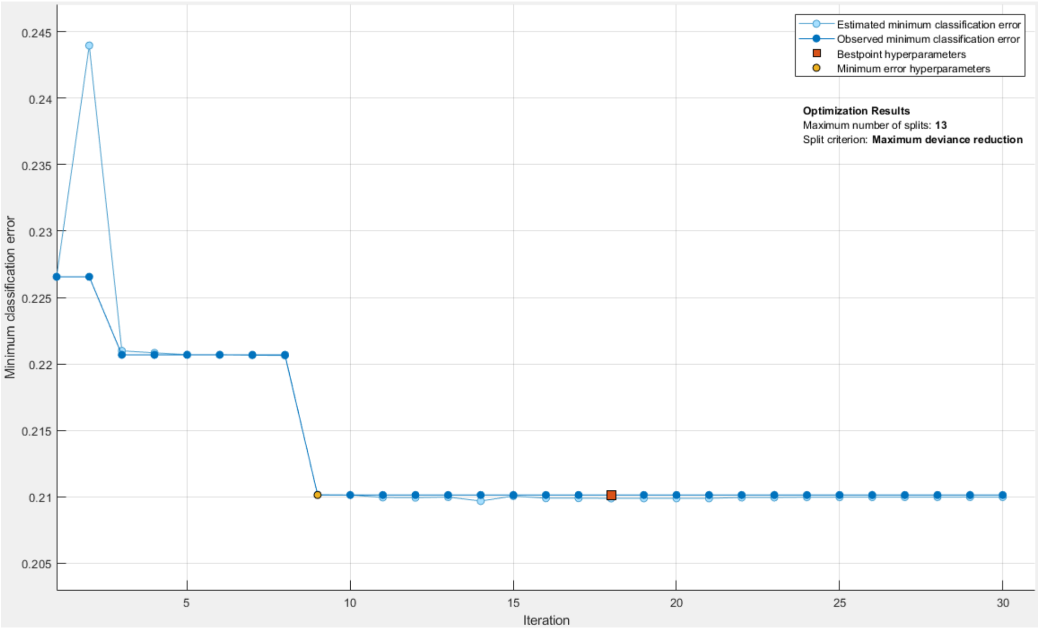

(i) DT, mean, original fs.

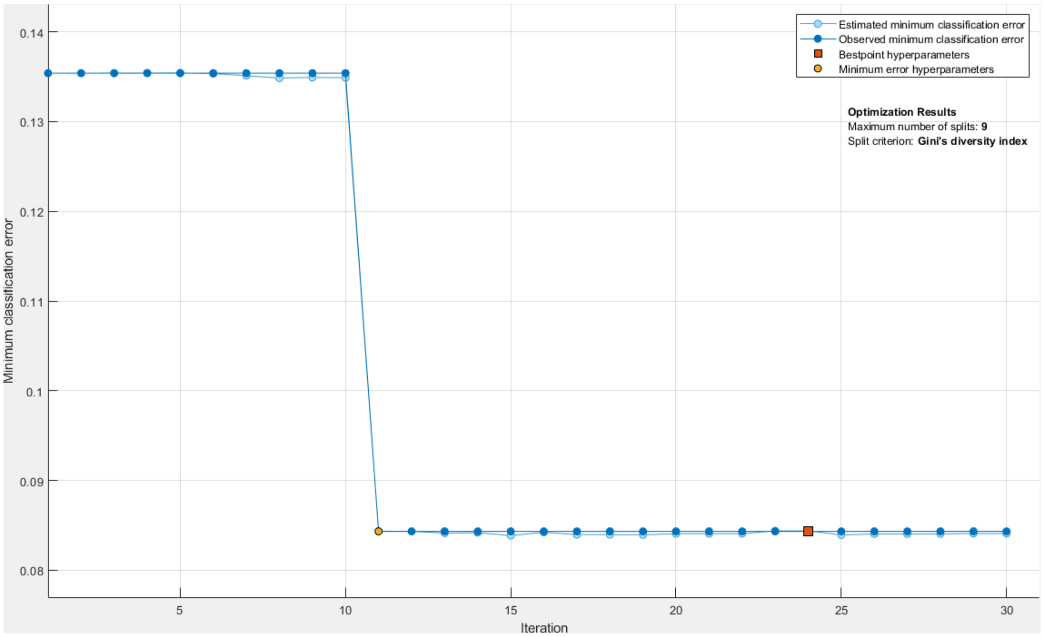

(j) DT, mean, LLT-based fs.

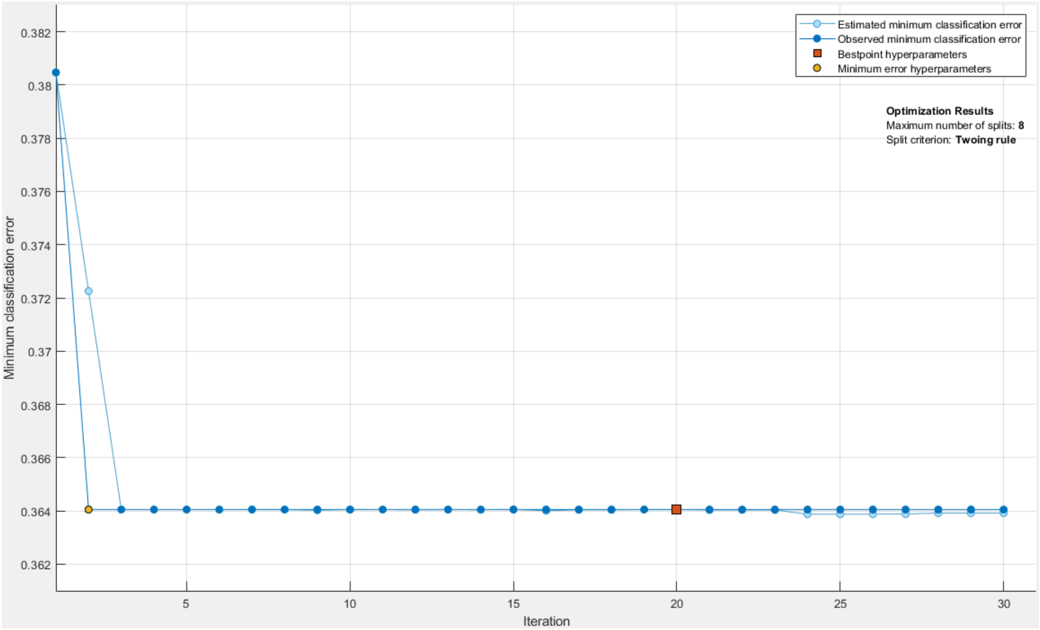

(k) DT, variance, original fs.

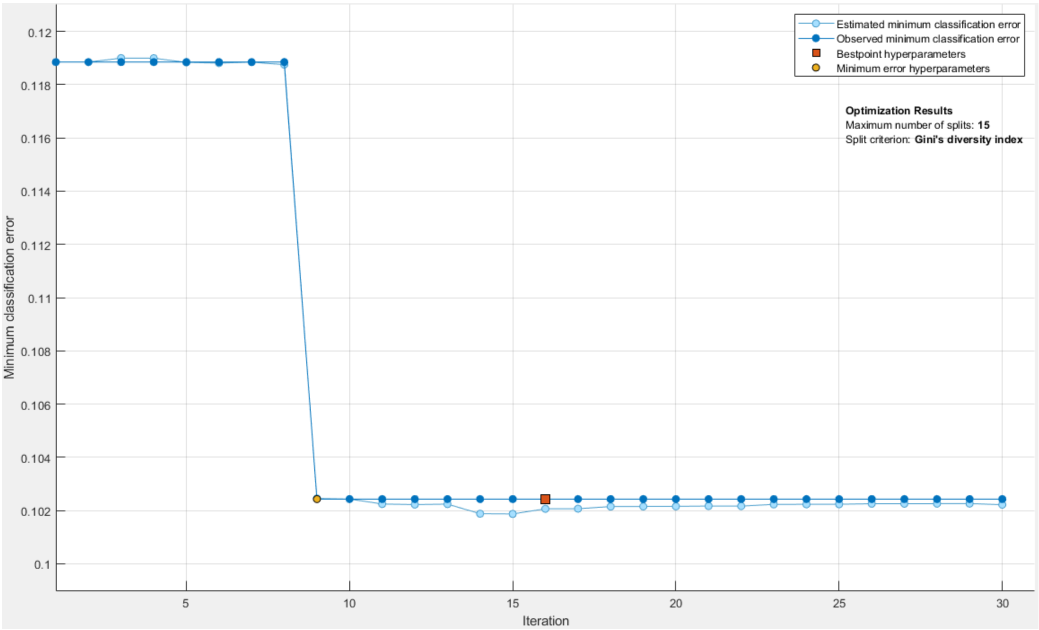

(l) DT, variance, LLT-based fs.

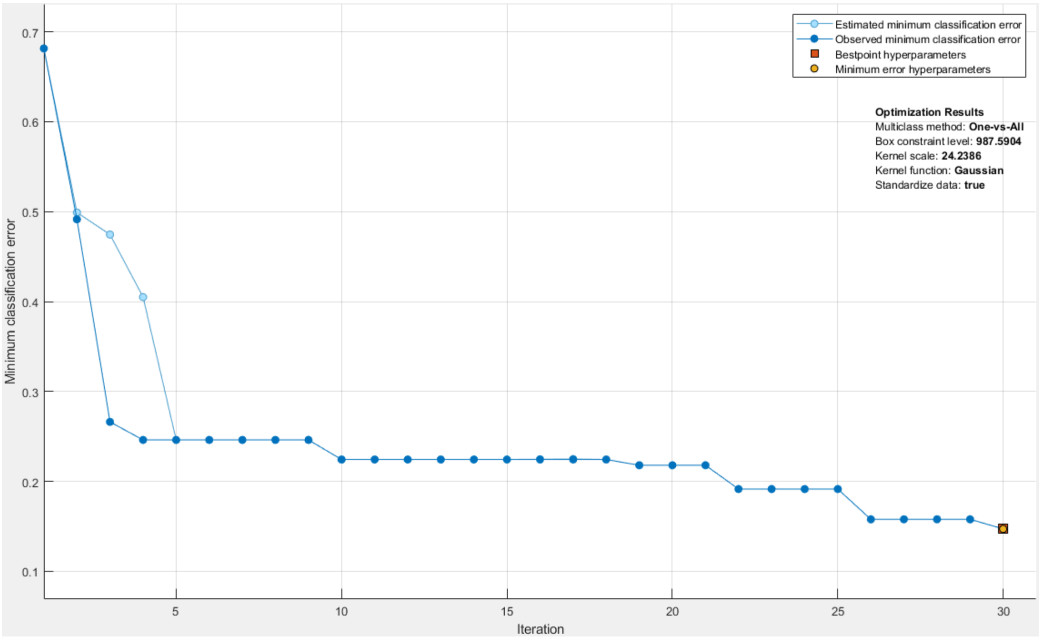

(m) SVM, mean, original fs.

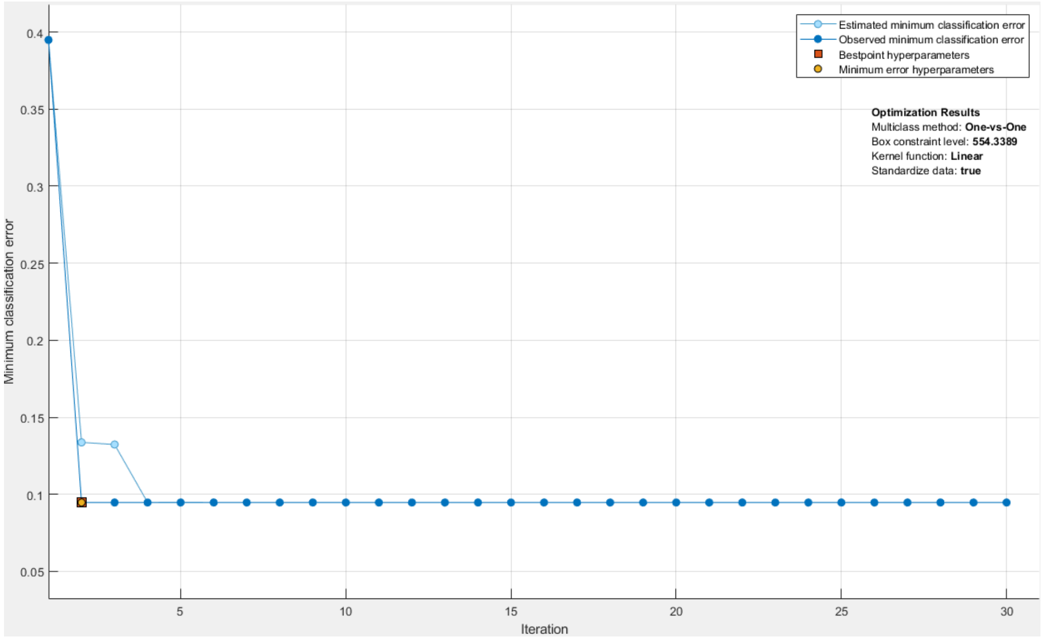

(n) SVM, mean, LLT-based fs.

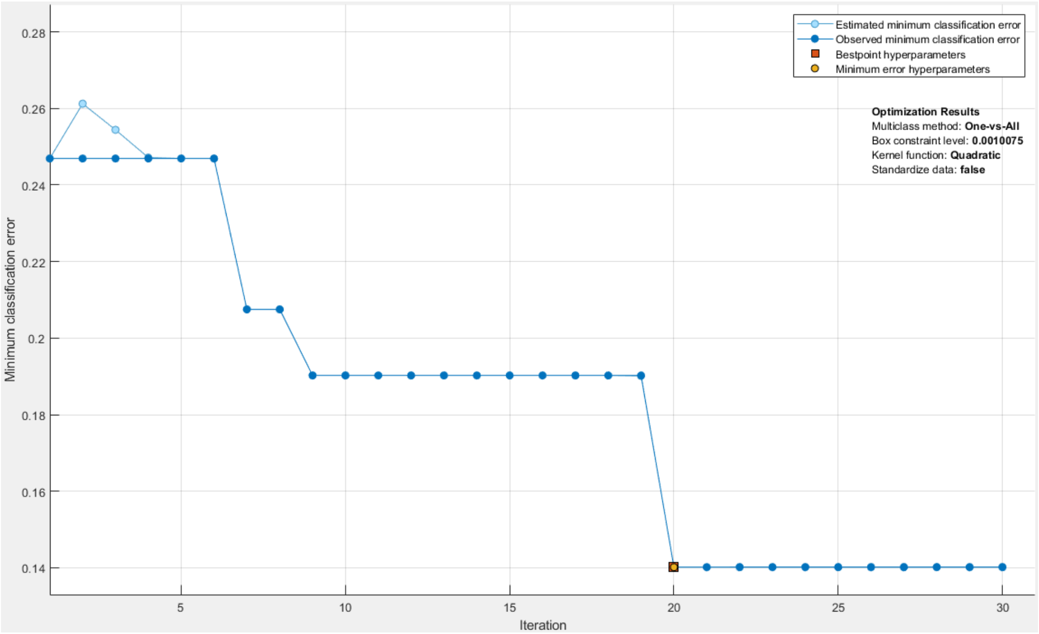

(o) SVM, variance, original fs.

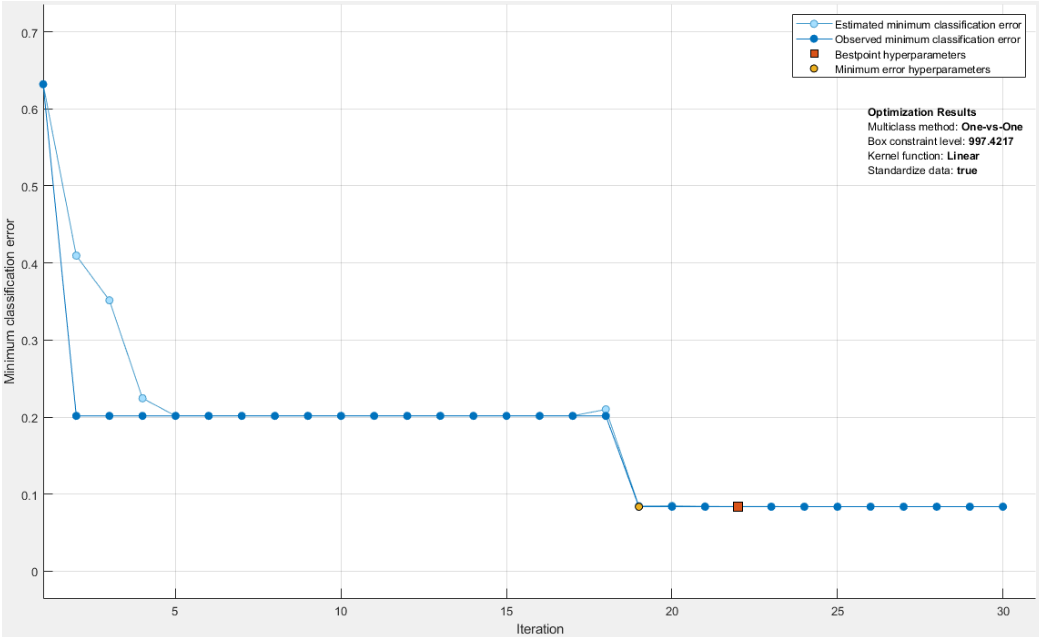

(p) SVM, variance, LLT-based fs.

Figure S2: Confusion matrices

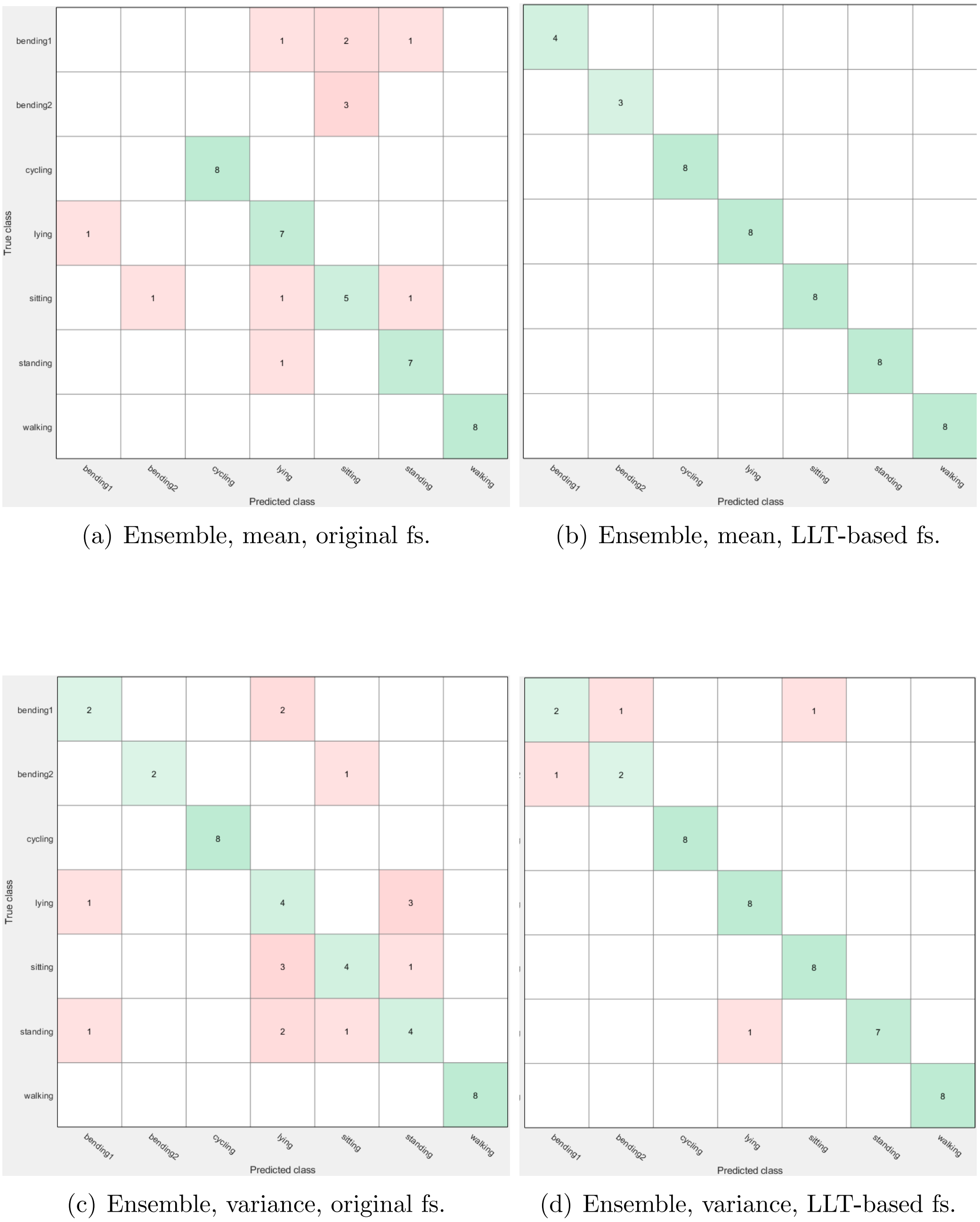

(a) Ensemble, mean, original fs.

(b) Ensemble, mean, LLT-based fs.

(c) Ensemble, variance, original fs.

(d) Ensemble, variance, LLT-based fs.

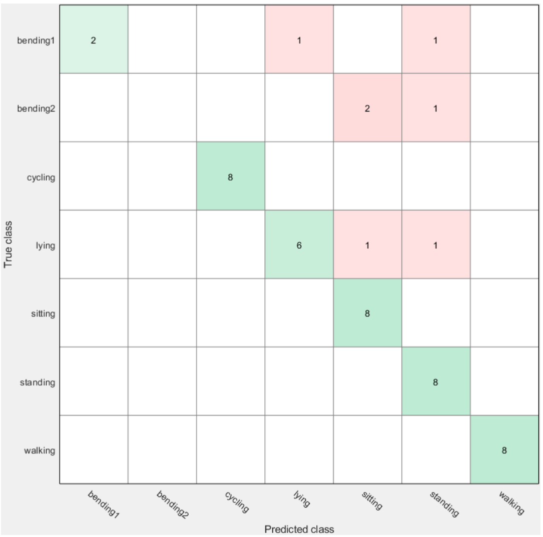

(e) KNN, mean, original fs.

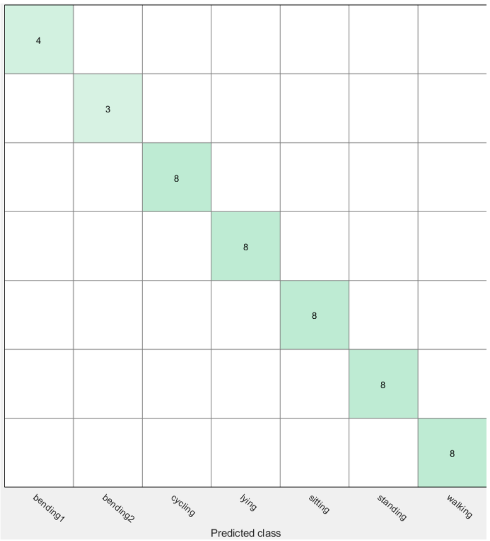

(f) KNN, mean, LLT-based fs.

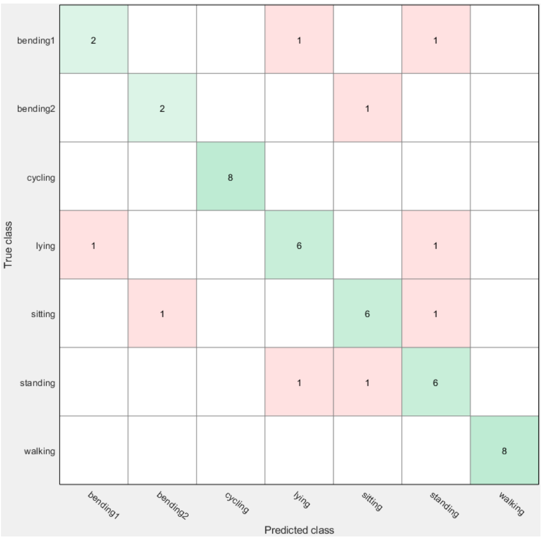

(g) KNN, variance, original fs.

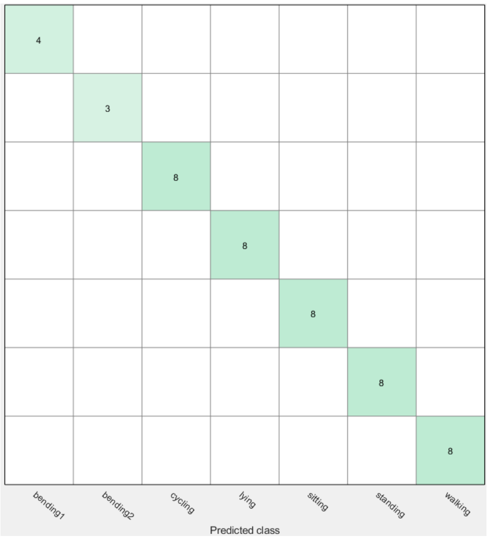

(h) KNN, variance, LLT-based fs.

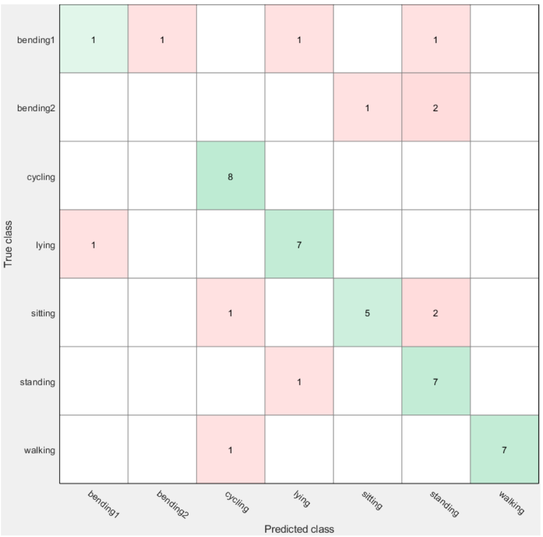

(i) DT, mean, original fs.

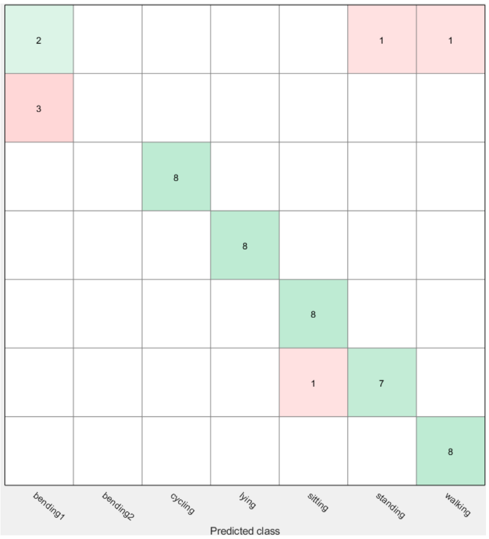

(j) DT, mean, LLT-based fs.

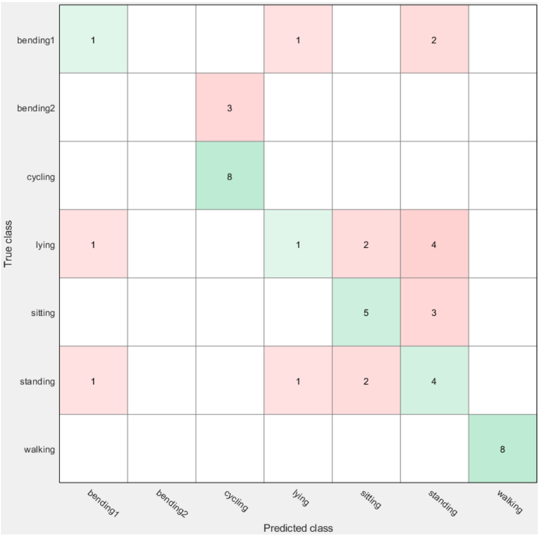

(k) DT, variance, original fs.

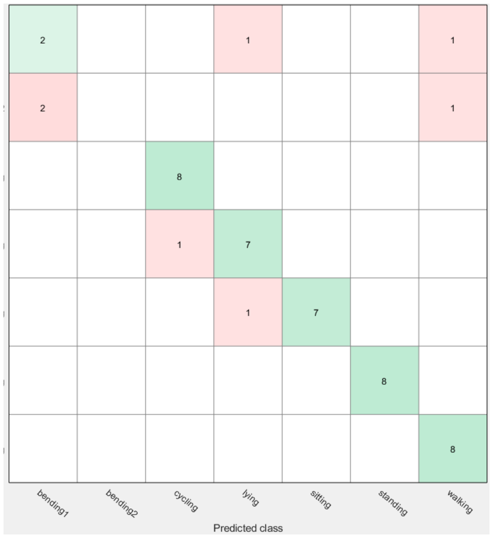

(l) DT, variance, LLT-based fs.

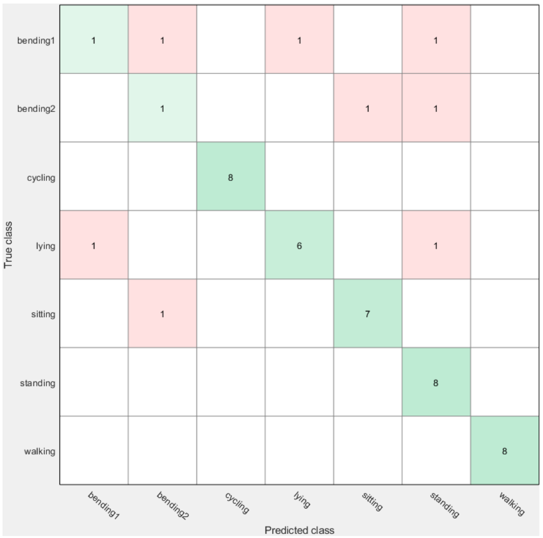

(m) SVM, mean, original fs.

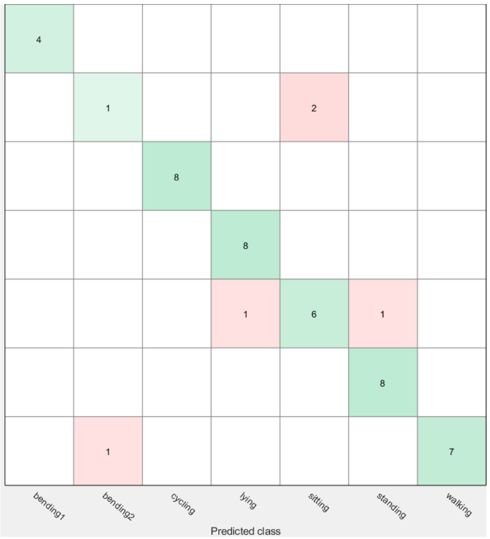

(n) SVM, mean, LLT-based fs.

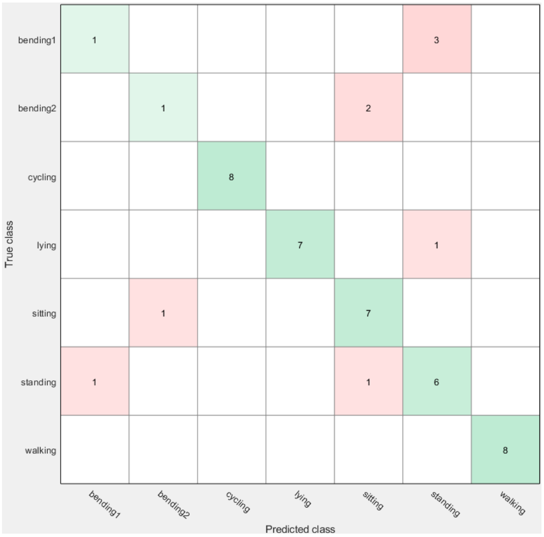

(o) SVM, variance, original fs.

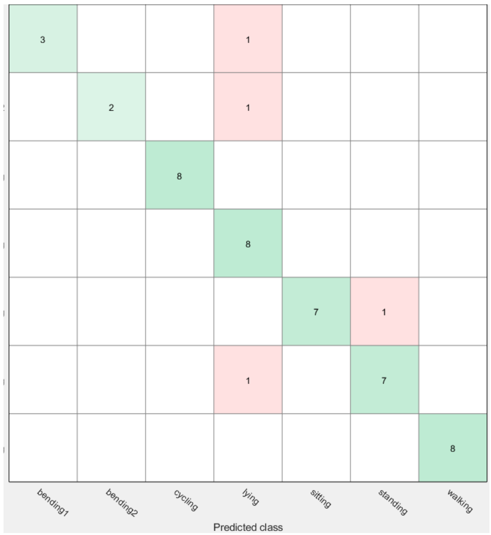

(p) SVM, variance, LLT-based fs.
